# RNA therapeutics in kidney diseases: prospects and current status

**DOI:** 10.1093/ckj/sfaf214

**Published:** 2025-07-11

**Authors:** Francesco Paolo Schena, Emanuela Pasculli

**Affiliations:** Department of Emergency and Organ Transplantation, University of Bari “Aldo Moro”, Bari, Italy; Schena Foundation, Bari, Italy; Schena Foundation, Bari, Italy; Department of Interdisciplinary Medicine, University of Bari “Aldo Moro”, Bari, Italy

**Keywords:** ASO, microRNA, RNA interference, short interfering RNA, therapy

## Abstract

In this narrative review, we summarize the current knowledge on RNA-based therapies used in rare and ultrarare disorders and congenital diseases in which the kidneys may be involved. In these therapies, RNA molecules are packaged into delivery vehicles to reach the desired target. We describe only drugs that have been approved or are under review for approval by the US Food and Drug Administration and/or the European Medicines Agency. We describe the potential therapeutic role of microRNA (miRNA) in Alport syndrome, polycystic kidney disease and renal cell carcinoma. Notably, large randomized clinical studies are required before these drugs can be introduced into clinical practice. The therapeutic effects of short interfering RNA molecules have been tested and evaluated in patients with various congenital or acquired diseases, such as primary hyperoxaluria, hereditary transthyretin amyloidosis, acute kidney injury after cardiovascular intervention or kidney transplantation (i.e. delayed graft function), and in individuals affected by hypercholesterolemia. In addition, synthetic antisense oligonucleotides have proven effective in patients with moderate or severe hypercholesterolemia who developed statin side effects, such as myalgia or rhabdomyolysis, and in individuals with amyloidosis. These new therapeutic approaches need to be validated through global clinical trials in which large patient samples can be enrolled. Nonetheless, some of these promising new approaches are currently undergoing evaluation for the treatment of common diseases, such as hypertension and diabetes, which are the main causes of chronic kidney disease.

## RNA INTERFERENCE: BIOLOGICAL MECHANISMS

**Figure 1:**
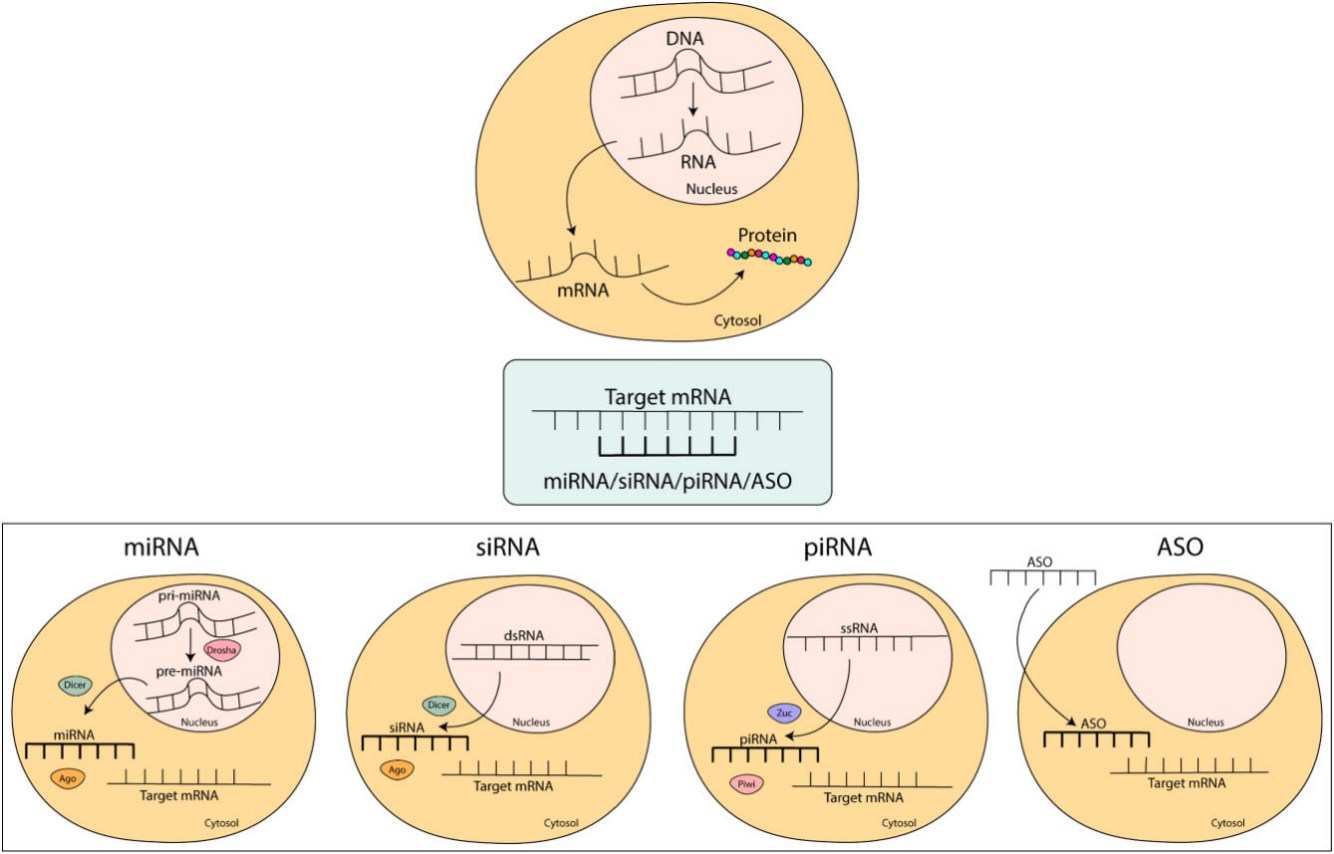
Interaction between a target mRNA and small single-stranded oligonucleotides. miRNA, siRNA, piRNA and ASO are small single-stranded oligonucleotides, both endogenous and exogenous, with a crucial role in gene expression regulation. They are able to bind to a target mRNA and induce gene silencing via mRNA cleavage or inhibition of translation. miRNA, siRNA and piRNA are part of the RNA interference pathway and are produced in the nucleus of cells and then undergoes different maturation steps. On the contrary, ASO is a synthetic oligonucleotide with the same role in gene silencing of the cellular ones.

Gene expression regulation is an intriguing field of study which has experienced tremendous development over the last few decades. The entire gene expression regulation is time specific and tissue dependent and must be finely regulated at multiple levels. In this particular context, the significance of endogenous non-coding messenger ribonucleic acids (RNAs), which play a crucial role in gene silencing, becomes apparent, facilitating the identification of RNA interference (RNAi) machinery [1]. The main components of this pathway (i.e. the RNAi machinery or RNAi pathway) are small non-coding RNAs (sncRNAs), such as microRNAs (miRNAs), short interfering RNAs (siRNAs), P-element Induced WImpy testis (PIWI)-interacting RNAs (piRNAs) and a clade of Argonaute (Ago) proteins, which, taken together, comprise the effector complex for gene silencing [2] (Fig. 1). RNAi action has been elucidated primarily at the posttranscriptional level, and is mediated through the targeting of messenger RNA (mRNA), via Watson–Crick base pairing, leading to gene downregulation [3]. The biogenesis mechanism and functional action of miRNAs and siRNAs share common features, while piRNAs differ in these aspects. Specifically, miRNAs and siRNAs originate from a double-stranded precursor molecule and subsequently undergo various maturation phases to achieve their single-stranded form, whereas the piRNA precursor is a single-stranded RNA. At the end of the maturation process, miRNAs and siRNAs possess a length of 18–25 nucleotides, while piRNAs remain longer, with 21–30 nucleotides [3, 4]. Furthermore, piRNAs form an effector complex in combination with a specific Ago clade protein, the PIWI protein, from which the name for this class of small endogenous RNAs (PIWI-interacting RNAs) is derived [5]. Because the PIWI protein is specifically identified in germline cells, piRNAs are tissue-specific and play a crucial role in gametogenesis and reproduction; in contrast, miRNAs and siRNAs are ubiquitous and have been identified in roughly all organisms and cell lines, but with different roles [3, 4]. Specifically, miRNAs are involved in the silencing of endogenous genes and siRNAs act primarily in defense of genome integrity against exogenous factors, such as transposable elements and viruses [6]. Regarding their mechanism of action, the key process is the loading of miRNAs or siRNAs into Ago proteins and the loading of piRNAs into PIWI proteins, to form the RNA-induced silencing complex (RISC). This effector complex is able to bind to its target, promoting its regulation. The Ago protein plays a key role in the RISC due to the Ago protein PAZ domain, which is involved in small RNAs (sRNAs)/target binding, and the Ago protein PIWI domain, with its inherent endonuclease activity, both of which contribute to the silencing function of the RISC [7]. The binding between these two single-stranded molecules (miRNAs or siRNAs and piRNAs) can have full or partial complementarity. Fully complementarity leads to mRNA cleavage and consequent degradation, mediated by cellular exonucleases. With partial complementarity, the RISC induces the inhibition of translation, either in the initiation phase or in the elongation phase, without affecting mRNA abundance in the cell cytosol [3]. These two mechanisms of action of the RNAi pathway have a considerable effect on various biological functions, such as the development, proliferation, differentiation and apoptosis of cells [8–10]. Dysregulation of the expression of the RNAi machinery components leads to the onset of different pathological conditions, such as cardiovascular diseases, kidney diseases, metabolic disorders and cancer [11].

## RNA INTERFERENCE: THERAPEUTIC APPROACHES

**Figure 2:**
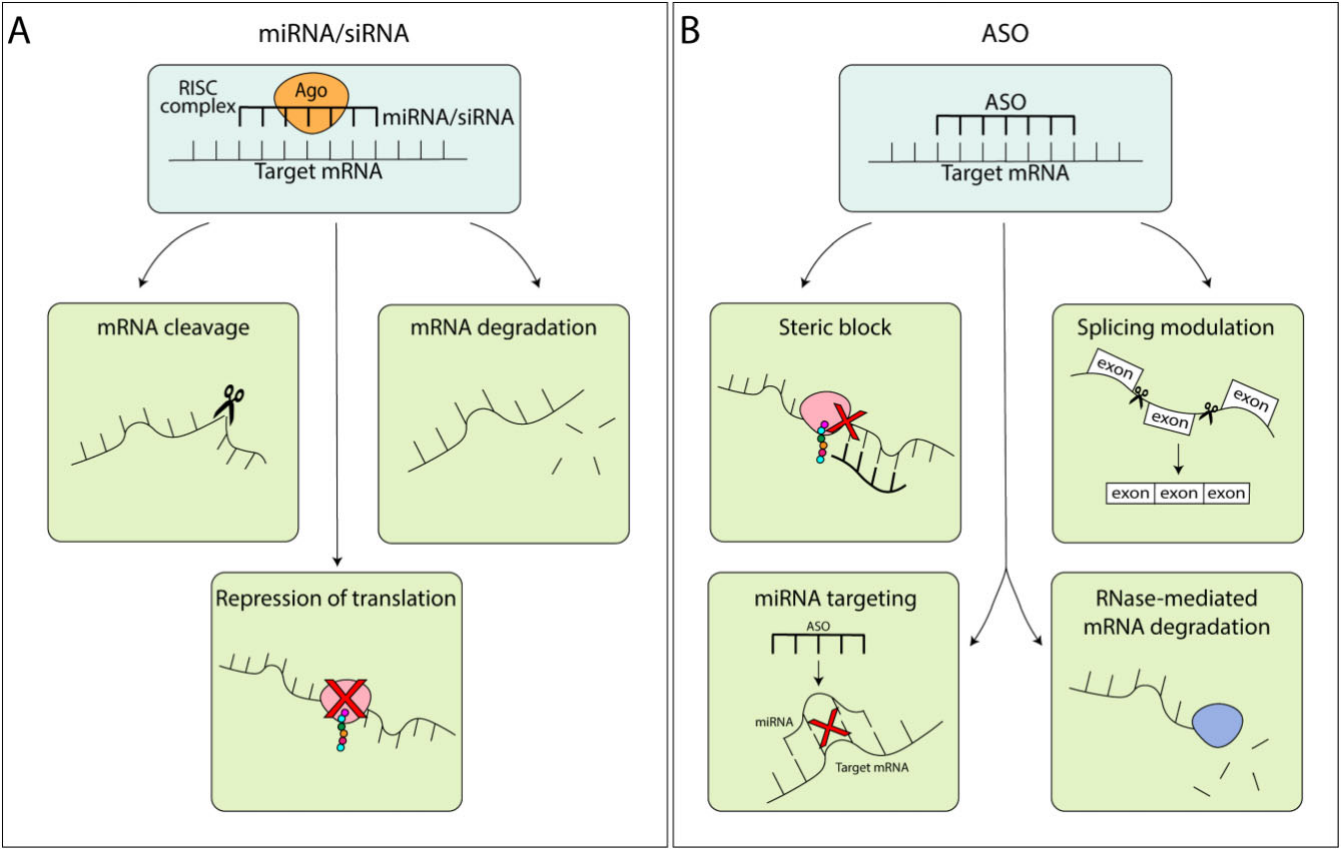
Mechanism of action of miRNA, siRNA and ASO. miRNA and siRNA are loaded into Ago protein in order to form the RISC complex, which is the effector complex, able to promote gene silencing via mRNA cleavage, mRNA degradation or repression of translation (**A**). ASO is able to directly bind target mRNA inducing its repression via steric block, splicing modulation, miRNA targeting or RNase-mediated mRNA degradation (**B**).

In an interesting development, emerging evidence points to the ability of these small RNAs to be exploited as novel therapeutic molecules in treating a wide range of diseases. Synthetic miRNAs and siRNAs have been designed to mimic the endogenous counterpart and engage in gene silencing, via mRNA cleavage or translational repression (Fig. 2A). These mimic molecules are present as double-stranded small molecules with chemical modifications that facilitate loading into cellular Ago proteins and subsequent guide strand selection. In this way, it is possible to not only regulate gene expression but also modulate the abundance of endogenous miRNAs or siRNAs, without impacting the biogenesis pathway [12, 13]. Another important strategy developed to assess gene regulation at the posttranscriptional level is embodied in antisense oligonucleotides (ASOs) (Fig. 2B). These synthetic oligonucleotides have a length ranging from 12 to 30 nucleotides and can bind to target RNA with perfect complementarity. Following target binding, they can induce cleavage and degradation or steric blocking, depending on the design of the ASO, the chemical modifications, the site of binding on the target and the function of the target. To achieve target RNA cleavage, ASOs exploit the enzymatic activity of RNase H, an endonuclease, that is ubiquitously expressed in cells. Another mechanism of action is steric blocking which promotes the inhibition of translation in the same manner as endogenous miRNAs and siRNAs. Another important mechanism of action employs the ability of ASOs to target endogenous miRNAs and siRNAs, in order to block their action and thus promote gene expression. Furthermore, some ASOs have been designed to modulate splicing mechanisms, and to alter the production of specific proteins [14, 15].

Starting with the biological mechanisms that regulate RNAi, from the biogenesis of different molecules involved in the process to the different modes of action, this review summarizes the current knowledge on the therapeutic approaches that have been developed to treat pathological conditions involving the kidneys. We describe only drugs that have been approved or are under review for approval by the US Food and Drug Administration (FDA) and/or the European Medicines Agency (EMA).

## MiRNA THERAPY

Several pharmaceutical companies are involved in the formulation of new RNA-based therapeutics and the main applications of these drugs are based on two approaches: (i) the improvement of miRNA function or miRNA replacement in individuals with diseases characterized by low levels of target miRNA or (ii) the inhibition or repression of target miRNAs using antagomiRs (anti-miRs). However, miRNA therapy faces several challenges, such as: (i) certain miRNAs may target multiple genes, thus causing deleterious side effects, and (ii) miRNAs exhibit opposing functions in different cells. Thus, various strategies are needed to selective deliver miRNAs to specific cell types involved in different diseases. To improve the *in vivo* stability of miRNA-based drugs and to protect these molecules using serum nucleases, biotech companies have developed RNA molecules with specific chemical modifications in their phosphodiester backbones. Several miRNA-based drugs are currently being evaluated in clinical trials or are under review for FDA and EMA approval.

Two clinical studies involving patients with Alport syndrome have been conducted (Table 1). In the first study (phase 1, NCT03373786, A Study of RG-012 in Subjects With Alport Syndrome), an anti-miR-21 (lademirsen; Sanofi Genzyme, Cambridge, MA, USA; SAR 339375, RG-012) was administered to the participants. This anti-miR is modified with 2′-fluoro substitutions (PS) and 2′-O-methyl phosphorothioate (2-O-MOE) to improve its safety. However, the results of this clinical trial have not been published [16]. The second clinical study was a phase 2 clinical trial with a randomized, double-blind, placebo-controlled study period followed by an open-label period. Lademirsen proved effective in adult Alport syndrome patients with estimated glomerular filtration rate (eGFR) of 35–90 mL/min/1.73 m^2^ [17]. The drug was administered subcutaneously at a dose of 110 mg, once a week. The trial was discontinued after a planned interim analysis because it showed no significant clinical benefits. During the clinical course of the study, patients developed critical side effects, such as headache, dizziness, metabolic/electrolyte alterations, anemia and respiratory tract infections. Furthermore, the eGFR slope was similar for the experimental and placebo groups with no significant differences observed. In conclusion, the drug was generally well tolerated, but the slowed progression of kidney fibrosis, observed in animal studies, was not achieved in humans. The main limitations of this clinical trial were the small number of patients enrolled as participants and the double-blind, randomized, placebo-controlled design of the trial which is difficult to realize and complete for a rare disease.

**Table 1:** List of mRNA blockers administered in patients with metabolic disorders and kidney involvement.

	Drug	Target	Trade name	Company	Disease/disorder
miRNA	Lademirsen	miR-21		Regulus Therapeutics-Sanofi	Alport syndrome
	RGLS 4326	miR-17		Regulus Therapeutics	Polycystic kidney disease
	MRX 34	miR-34a		Mirna Therapeutics	Renal cell carcinoma
siRNA	Lumasiran	HAO1	Oxlumo	Alnylan	Primary hyperoxaluria
	Nedosiran	HLDH	Rivfloza	Dicerna Alnylan	Primary hyperoxaluria
	Patisiran	TTR	Onpattro	Alnylan	TTR amyloidosis
	Vutrisiran	TTR	Amvuttra	Alnylan	TTR amyloidosis
	Teprasiran	p53	QPI-1002	Quark Novartis	AKI, DGF
	Inclisiran	PCSK9	Leqvio	Alnylan Novartis	Hypercholesterolemia
	Cemdisiran	C5 complement		Alnylan Pharmaceutics	IgAN
ASO	Mipomersen	apoB-100	Kynamro	Alnylan-Genzyme	Hypercholesterolemia
	Inotersen	TTR mRNA	Tegsedi	Ionis Pharmaceutics	TTR amyloidosis
	Sefaxersen	Complement Factor B	Ionis-FB-LRx	Ionis Pharmaceutics	IgAN

DGF, delayed graft function; HAO1, glycololate oxidase; HLDH, hepatic lactate dehydrogenase.

A phase 1 clinical trial study (NCT04536688, A Study of RGLS4326 in Patients With Autosomal Dominant Polycystic Kidney Disease) of another miRNA-based therapy was conducted in patients with autosomal dominant polycystic kidney disease (ADPKD), who received RGLS4326, an anti-*miR-17* developed by Regulus Therapeutics (San Diego, CA, USA). Notably, miRNA-17 has been found to be highly expressed in the renal tissue of ADPKD patients. Nonclinical pharmacologic studies using anti-miR-17 oligonucleotides have been conducted in mouse models of PKD by effectuating miRNA inhibition and downregulating *PKD1* and *-2* genes [18]. Lee *et al*. conducted an open-label, adaptive design, dose-ranging, phase 1b clinical trial to evaluate the safety of RGLS4326, and the pharmacodynamics and pharmacokinetics in patients with ADPKD [19]. Nine patients with a mean eGFR of 49 mL/min/1.73 m^2^ were enrolled in the trial and each patient received one of the three established doses (1 mg/kg, 0.3 mg/kg, or 0.1 or 0.5 mg/kg administered every-other-week for a total of four doses) via subcutaneous injection. The RGLS4326 drug was administered over a 6-week period and was well tolerated with no serious adverse events. A reduction in the rise of policystin-1 (PC1) and PC2 levels was observed at the end of the study. However, other studies are needed to further evaluate and validate RGLS4326 as a proven therapeutic approach in ADPKD patients.

Finally, the miR-34 mimic (MRX34; miRNA Therapeutics, Austin, TX, USA) has been administered to patients with advanced solid tumors, including refractory renal cell carcinoma to standard treatment. The miR-34a is a key regulator of tumor suppression controlling the expression of several targets involved in the cell cycle (c-MYC, E2F, CDK4 and CDK6), apoptosis (BCL2 and SIRT1) and tumor-associated invasion (c-MET). MRX34 is a 23-nucleotide-long, double-stranded miRNA that resembles the endogenous miR-34a duplex and is encapsulated in lipid nanoparticles for intravenous administration. MRX34 interferes with target gene translation by binding to complementary sequence in the 3′-untranslated region of target mRNA. In a standard 3 + 3 dose escalation trial into which 47 adult patients with various solid tumors were enrolled [20, 21], MRX34 was administered intravenously twice weekly for 3 weeks in 4-week cycles (phase 1 clinical trial, NCT01829971, A Multicenter Phase I Study of MRX34, MicroRNA miR-RX34 Liposomal Injection). Dexamethasone premedication was required to manage infusion-related adverse events. This clinical trial successfully demonstrated perfect regulation of miR-34, and treatment with MRX34 was associated with acceptable levels of safety, with evidence of antitumor activity in a subset of patients with refractory advanced solid tumors.

## SiRNA THERAPY

siRNAs are short (20–27 nucleotides long) double-stranded RNAs that target and degrade mRNA in a sequence-specific manner. They have low bioavailability due to their large size (13–14 kDa) and anionic charge. Nanocarrier-encapsulated drugs with low serum protein absorption, high cellular penetrance and high rate of escape from endosomal trapping have been developed to improve siRNA delivery. Thus, two strategies have been used to improve the availability of siRNA drugs: (i) chemical modifications involving the replacement of the phosphodiester bond with a phosphorothioate (PS) backbone, comprising mainly partially PS-modified siRNA molecules and (ii) delivery systems, which involve the formulation of siRNA into nanocarriers for cell transfection and conjugation of siRNA to a targeting ligand [22]. Lipid nanoparticles have been successfully used in the delivery strategy of siRNA therapy. They are encapsulated within pegylated lipid nanoparticles to prevent degradation and to reduce aggregation, opsonization and reticuloendothelial system clearance. Furthermore, these large nanoparticles may pass through the fenestrated endothelium or target specific cells [23]. This strategy has been used to deliver patisiran which is the first siRNA drug approved by FDA [24]. Other strategies of siRNA delivery involve the conjugation of siRNAs with micelles, dendrimers, niosomes, metallic nanoparticles, human serum albumin-based nanoparticles, oligonucleotide nanoparticles and cationic transfection agents [25]. siRNAs have the potential to provide long-lasting effects with a single dose, thus improving patient compliance.

Recently, bioconjugates containing targeting ligands have been used to transport siRNAs. Glycoproteins terminating with N-acetylgalactosamine (GalNAc) sugars are bioconjugates that bind with high affinity to asialoglycoprotein receptors, which are highly expressed on hepatocytes. This mechanism has been used to deliver givosiran [26] and lumasiran [27]. Other GalNAc conjugates are vutrisiran, nedosiran, inclisiran and fitusiran [23]. siRNA drugs can also be conjugated to cationic peptide moieties (penetratin) or lipophilic moieties (cholesterol). The siRNAs described in this review are presented in Table 1.

Lumasiran (Oxlumo) and nedosiran (Rivfloza) are two siRNA drugs, approved by the FDA and the EMA, which are administered to subjects with primary hyperoxaluria type 1 (PH1) who have a deficiency in hepatic alanine glyoxylate aminotransferase (AGXT) enzyme and, consequently, high levels of oxalate production [28]. The presence of mutations in *AGXT* gene is responsible for the reduced production of the enzyme that converts glyoxylate and alanine into pyruvate and glycine. Consequently, accumulated glyoxylate is converted into oxalate, which is normally eliminated via the kidneys, but its overproduction and high blood concentration lead to deposition of calcium oxalate stones in the kidneys and other tissues. Progressive accumulation of calcium oxalate in the kidneys causes chronic kidney disease (CKD), a gradual increase in serum creatinine levels and end-stage kidney disease. Currently the primary treatment for hyperoxaluria is based on hydration and urine alkalinization. Recently, considerable success has been achieved using siRNA therapy to treat this rare metabolic disease [29]. Lumasiran (Oxlumo; Alnylam Pharmaceuticals, Cambridge, MA, USA) and nedosiran (Rivfloza; Dicerna Pharmaceuticals, Lexington, MA, USA), which target hepatic glycolate oxidase and lactate dehydrogenase, respectively, have been successfully administered to patients with PH1 who subsequently experienced reduced hepatic expression of glycolate oxidase and lactate dehydrogenase, reduced production of hepatic oxalate and reduced urinary levels of oxalate. The lumasiran dose (3 mg/kg body weight per month subcutaneously) induced maximal reduction in the urinary oxalate levels. The indication is as follows: loading dose: 3 mg/kg subcutaneously once a month ×3 doses, and maintenance dose: 3 mg/kg every 3 months. Lumasiran was also administered to PH1 patients with end-stage kidney disease who had undergone combined liver–kidney transplantation because the oxalate production had not completely normalized in some patients who underwent this procedure. Therefore, monitoring of oxalate levels must be continued after transplantation [30]. The nedosiran dose for patients with a body weight of <50 kg is 128 mg (prefilled syringe, 0.8 mL), injected subcutaneously once monthly and 160 mg (prefilled syringe, 1 mL) once monthly for individuals with a body weight of more than 50 kg. The drug has also been successfully administered to patients with PH2 and is under evaluation for use in PH3 patients [31]. A recent systematic review and meta-analysis has shown that early intervention in PH patients has benefits for managing kidney function, and it is possible to reverse the renal effects of hyperoxaluria. Furthermore, a high dose and long therapy duration may improve clinical outcomes [32].

Patisiran (Onpattro; Alnylam Pharmaceuticals, Cambridge, MA, USA) has been administered to patients with hereditary transthyretin (TTR) amyloidosis, an autosomal dominant disease in which the accumulation of misfolded TTR proteins in the body causes progressive neuropathy, cardiomyopathy, CKD and organ failure. The siRNA component of patisiran is packaged in liver-specific lipid nanoparticles and silences the mutant *TTR* gene, thus reducing the production of TTR proteins and their deposition in the organs. Lipid nanoparticles have a high affinity for the apolipoprotein E (Apo-E) receptors of the hepatocytes which are the main producers of TTR [33]. In phase 3 of the APOLLO study of patrisan, into which 225 patients were enrolled and randomly assigned to a treatment regimen, patisiran therapy reduced the serum TTR levels in 81% of the patients over 18 months of treatment. The drug was generally well-tolerated, with a low percentage of adverse events such as cutaneous reaction at the site of injection, upper respiratory tract infections and peripheral edema [34]. The recommended dose for patisiran is 0.3 mg/kg of body weight delivered intravenously over 80-min once every 3 weeks, preceded by premedication (corticosteroids, antihistamines and acetaminophen). In a retrospective study, involving 103 patients, 16.5% of them had CKD at the time of TTR amyloidosis diagnosis, while 16.1% had proteinuria. Patisiran administration induced progressive remission of the nephrotic syndrome in a young patient [35].

Vutrisiran (Amvuttra, Alnylam Pharmaceuticals, Cambridge, MA, USA) is another siRNA drug approved by the FDA and the EMA for therapy in patients affected by TTR amyloidosis. Vutrisiran is chemically stabilized and delivered via a third generation GalNAc platform. Two phase 3 clinical trials of vutrisiran (HELIOS-A and HELIOS-B) have been conducted by the Alnylan Pharmaceuticals and a large number of patients with TTR were enrolled. In the first study (HELIOS-A), participants received either vutrisiran at a dose of 25 mg, administered subcutaneously every 12 weeks or patisiran at a dose of 0.3 mg/kg administered intravenously every 3 weeks for 18 months [36]. The placebo group was an external APOLLO placebo group. The clinical trial results showed improvement in the N-terminal pro-brain natriuretic peptide (NT-proBNP) serum levels, and echocardiographic parameters of participants in comparison with placebo group. In the HELIOS-B study, a sizable number of patients with TTR amyloidosis (655 individuals) were randomized to either vutrisiran or placebo treatment [37]. The drug was administered subcutaneously every 3 months during a 3-year treatment period. This clinical trial was completed recently, and the results proved that the siRNA drug lowers the levels of the circulating TTR protein, and leads to a reduction of death risk from any cause, including cardiovascular events. Furthermore, vutrisiran provides benefits to patients in the early stages of TTR amyloidosis.

Teprasiran (QPI-1002), developed by the Quark Pharmaceuticals (Ness Ziona, Israel) and licensed by Novartis (Basel Switzerland), is engineered for the prophylactic treatment of acute kidney injury (AKI) following cardiovascular intervention or kidney transplantation (delayed graft function). A pro-apoptotic transcription factor, *p53*, is activated in AKI and teprasiran targets the mRNA of this factor. This drug is intravenously administered as a naked siRNA with no delivery system or conjugated to a targeting ligand; it is cleared by glomeruli and reabsorbed via endocytosis in the proximal tubular cells involved in AKI. The therapeutic effect of teprasiran (10 mg/kg body weight in phosphate buffered saline) administered as a single intravenous bolus after 4 h of discontinuation of cardio-pulmonary bypass, and after the last coronary anastomosis for off-pump surgery, was evaluated in more than 300 patients [38] in a prospective, multicenter, double-blind, randomized controlled phase 2 trial which revealed that teprasiran lowers the incidence of AKI and ameliorates the duration and severity of AKI. The drug has also been administered to patients undergoing kidney transplantation from cadaveric donors as a prophylaxis of delayed graft failure [39]. These patients received a single dose of QPI-1002 (10 mg/kg body weight) intravenously 30 min after circulatory reperfusion of the transplanted organ. The drug was well tolerated, and a relative reduction in delayed renal function was observed. Furthermore, QPI-1002 reduced the need for dialysis in recipients of expanded criteria donor kidneys; however, definitive results have not yet been released.

Inclisiran (Leqvio), originally developed by Alnylam Pharmaceuticals and subsequently licensed by Novartis (Basel, Switzerland) is an siRNA drug that targets proprotein convertase subtilisin/kexin type 9 (*PCSK9*) mRNAs, thus achieving a sustained reduction in low-density lipoprotein cholesterol (LDL-C) levels in patients with hypercholesterolemia. The drug utilizes the enhanced stabilization chemistry (ESC)-GalNAc delivery platform with high uptake in hepatocytes following subcutaneous administration. Reduction in LDL-C serum levels was achieved at 30 days of therapy. A few years ago, a biopharmaceutical company launched the ORION program, which comprised several randomized clinical trials that have been conducted, first, in patients with familial hypercholesterolemia and then, in individuals with atherosclerotic cardiovascular disease (ASCVD) who had received the maximum dose of statins and/or ezetimibe for at least 30 days before screening for the trial [40]. The clinical trial outcomes demonstrated that inclisiran, when administered at a dose of 300 mg on Days 1, 90, 270 and 450, decreased PCSK9 levels by 60% and reduced LDL-C by 50% compared with the placebo group in 1.5 years. ASCVD patients with normal, mild, moderate or severe renal impairment were enrolled in the ORION-7 renal study and phase 2 of the ORION-1 study, and the pharmacodynamic effects and the safety profile of inclisiran were similar in patients with normal and impaired renal function. Therefore, a dose adjustment of the drug is not required for such patients [41]. Inclisiran was recently approved by the FDA and the EMA. However, a recent meta-analysis of data from five randomized clinical trials shows that no significant decrease in cardiovascular end points was observed in patients treated with inclisiran vis-à-vis placebo [42]. Prolonged exposure to inclisiran has been demonstrated to be safe and well tolerated in a broad group of patients with hypercholesterolemia, based on results from a constellation of clinical trials (seven ORION studies) [43]. The mean duration of exposure to inclisiran was 2.8 years in more than 3500 patients, with a small number treated beyond 5 years; however, treatment-emergent adverse events, including serious events, occurred at similar rates vis-à-vis treatment with a placebo.

A systematic review and pooled analysis of available studies have demonstrated that inclisiran has favorable effects on serum lipid levels and an acceptable safety profile [44]. A recent review focusing on novel therapies for achieving the recommended target LDL-C serum levels recommend the use of early aggressive inclisiran therapy after coronary artery bypass surgery in patients with statin intolerance [45]. Furthermore, Ray *et al*. demonstrated that twice-early administration of inclisiran in patients enrolled in a prospective long-term study (ORION-3 and phase 2 ORION-1) provided sustained reduction in serum LDL-C and PCSK9 concentrations. The drug was well tolerated for over 4 years of therapy in an extension study [40].

## ASO THERAPY

An ASO is formed by a single strand of 12–22 oligodeoxynucleotides that are complementary to the target mRNA sequence of a specific gene. The binding of ASOs to tagged mRNA induces the inhibition of a translational process in the ribosomal complex and the induction of RNase H, which cleaves the 3′-O-P-bond of an RNA molecule. This mechanism has the complete specificity for the target gene. Later, ASOs have been modified by replacing the non-bridging oxygen molecules in the phosphate backbone with sulfur molecules, thus improving ASO resistance to nuclease activity. This second generation of ASOs supports RNase H activity and enhances nuclease resistance and RNA affinity. In conclusion, ASOs act in two different ways: (i) via the formation of the RNA: DNA heteroduplex with complementary mRNA, in which the RNA strand is recognized and cleaved by RNase H; and (ii) via steric blocking of the splicing and translation processes that constitute pre-mRNA binding.

Fourteen ASO drugs have been approved by the FDA and/or the EMA as of March 2024. These new drugs are designed to treat rare disorders or diseases. We focus only on two ASOs (Table 1). The first is mipomersen (Kynamro), a second-generation ASO, that targets the mRNA of apolipoprotein B-100 (apoB-100) to prevent its translation in the liver, thus reducing the production of LDL-C, very low-density lipoprotein cholesterol (VLDL-C) and lipoprotein(a) [Lp(a)]. This drug has been administered to patients with homozygous familial hypercholesterolemia who are at high risk of atherosclerotic cardiovascular disease and CKD. It can also be administered to patients with moderate or severe hypercholesterolemia who are experiencing statin side effects, such as myalgia or rhabdomyolysis, which are accompanied by a substantial release of muscle-derived enzymes into the bloodstream that can accumulate in renal tubules and thus cause kidney damage. Mipomersen has been administered subcutaneously once a week with a preformulated syringe containing 200 mg/mL of the drug in phase 1, 2 and 3 clinical trials [46]. Two recent meta-analyses [47, 48] evidenced a significant reduction in serum LDL-C levels, total cholesterol, Lp(a) and triglycerides with no significant effects on serum HDL-C levels. Adverse events in patients treated with mipomersen were as follows: increased hepatic enzymes, hepatic steatosis, flu-like symptoms and reaction at the injection site (pain, erythema and pruritus). The drug dose was reduced by 200 mg to 70 mg three times weekly to lessen these adverse effects and improve tolerability and adherence. However, because of these limitations, the drug has been approved by the FDA only for patients with homozygous familial hypercholesterolemia. Recently, mipomersen has been taken off the market due to its hepatotoxicity risk.

The second ASO we wish to discuss is inotersen (Tegsedi), which is administered to adult patients affected by polyneuropathy due to the deposition of hereditary TTR amyloidosis, a rare disease. This type of amyloidosis involves many organs, including the kidneys. This ASO drug causes degradation of mutant and wild type TTR mRNAs by the binding to the TTR mRNAs; thus, it reduces serum TTR protein levels and TTR deposits in organs including the kidneys. Inotersen is administered, subcutaneously, via injection at a dose of 284 mg, once a week on a fixed day [49]. Glomerulonephritis is an adverse event that may occur in 3% of patients receiving inotersen. Therefore, this ASO is advised only for TTR amyloidosis patients with a daily proteinuria <1 g/day. Inotersen-associated thrombocytopenia has been reported [50]. The key strategy to prevent this adverse-related side effect is monitoring the platelet count. Moreover, caution should be used in patients who receive antiplatelet drugs or anticoagulants. Liver is the site where the ASOs accumulate, therefore, it is recommended to monitor hepatic transaminases and total bilirubin at base line and every 4 months during the ASO therapy. Finally, one of inotersen's reported adverse drug events is glomerulonephritis; therefore, this ASO should not be initiated in patients with proteinuria >1 g/day. Recently, a case of focal segmental glomerular sclerosis (FSGS) complicating therapy with inotersen has been described [51].

The APOL1-mediated kidney disease occurs in African Americans, carrying two high-risk alleles which confer an increased risk of FSGS and end-stage kidney disease [52]. APOL-1 is the therapeutic target to treat this clinical entity. APOL1 ASO, developed by Ionis Pharmaceuticals, has been selected as candidate agent and a phase 1 study (NCT04269031, A First-in-human Study to Assess the Safety, Tolerability, Pharmacokinetics and Pharmacodynamics of AZD2373 After Single Dose Administration in Healthy Male Subjects of African Ancestry) has been carried out to evaluate and assess the pharmacokinetics of the ASO at escalating single doses (ION532). A clinical trial aimed to evaluate the efficacy, safety and pharmacokinetics of oral APOL1 inhibitor (VX-147) is ongoing in patients with FSGS and APOL1 G1/G1, G2/G2 or G1/G2 genotypes (NCT04340362, Phase 2a Study of VX-147 in Adults With APOL1-mediated Focal Segmental Glomerulosclerosis) [53].

ASO drugs are promising pharmaco-agents with a certain hepatic and renal toxicity, and occurrence of hypersensitivity reactions. Therefore, a better understanding of the risk will be attained and the benefit to risk ratio for patients need to be considered before to decide ASO treatment.

## RNA-BASED THERAPIES IN HUMAN GLOMERULONEPHRTIS

The current management of human glomerulonephritis is based on the use of non-specific immunosuppressive and/or anti-inflammatory drugs such as corticosteroids, cyclophosphamide, mycophenolate and calcineurin inhibitors. These drugs, if not correctly administered, may have a relatively high toxicity. However, the clinical benefits of these drugs are supported by numerous retrospective observational studies and randomized clinical trials carried out in patients with primary or secondary glomerulonephritis.

Recent advancements in RNA technology have provided an efficient administration of oligonucleotides (siRNA, ASO) in patients affected by glomerulonephritis (Table 1).

Cemdisiran is an siRNA, covalently linked to an N-acetylgalactosamine ligand, that reduces the liver production of C5 mRNA. It has been tested in biopsy-proven immunoglobulin A nephropathy (IgAN) patients in phase 2 randomized, double blind, placebo-controlled trial, in which 20 participants received subcutaneous cemdisiran 600 mg every 4 weeks in combination with standard therapy (angiotensin-converting enzyme inhibitor) [54]. Using a mixed effect model for repeated measures, the change from baseline in 24-h urine protein creatinine ratio was –31.4% at Week 32 compared with placebo group (–0.42%). The efficacy of cemdisiran was documented by a reduction from a baseline in hematuria grade in 77.3% of drug-treated patients compared with placebo group (22.2% of participants). The cemdisiran-treated group of patients had a mean percentage change in serum C5 levels of –98.7% from baseline at Week 32 compared with –25.2% in the placebo group. Reduction in serum complement alternate pathway (–48.1%) was also observed. At the fourth week, C5 reduction preceded the week reduction in proteinuria. In conclusion, this phase 2 study showed a constant reduction of daily proteinuria. Limitations of this study are (i) the complement study and the measurement of proteinuria were not done after cemdisiran cessation; and (ii) proteinuria did not achieve a value <0.5 g/day which is not dangerous for the progression of renal damage.

Other RNAi therapeutics, not yet approved by the FDA and EMA, have been recently developed by Biocompanies. SangeneBio (Woburn, MA, USA) has announced phase 2 clinical trials to target the C3 complement in complement-mediated renal diseases, including IgAN, C3 glomerulopathy and immune complex–mediated membranoproliferative glomerulonephritis (https://www.sanegenebio.com/98/194). Preliminary data have demonstrated a dose-dependent and sustained C3 reduction after a single subcutaneous injection of SGB-9768. This siRNA achieves great target protein knockdown and longer duration effect of the siRNA.

The complement system has a protective role against infections and prolonged complement inhibition may increase the potential risk of infections (i.e. meningococcal infections). Therefore, the decision to start with an anti-complement therapy should be well evaluated and during the treatment with complement inhibitors, antibiotic prophylaxis or vaccines should be associated and the balance between benefits and side effects should be well evaluated.

Sefaxersen (RO7434656) is a GalNAc-conjugated 2-MOE antisense oligonucleotide that has been synthesized and purified by Vetter Development services USA (Stokie, IL, USA) and packaged, labelled and distributed by Ionis Pharmaceuticals, Inc (Carlsbad, CA, USA). This ASO targets the complement factor B (CFB) mRNA, reduces the amount of CFB transcript and subsequently lowers the production of CFB protein [55]. An exploratory, single-arm, multinational open label phase 2 study (NCT04014335, A Study to Evaluate the Effectiveness and Safety of IONIS-FB-LRx, an Antisense Inhibitor of Complement Factor B, in Adult Participants With Primary IgA Nephropathy) recruited 19 IgAN patients who completed the clinical study [56]. The drug demonstrated an acceptable safety profile with no SAEs, reduced CFB serum levels and proteinuria improvement. Recently, it has been proposed a phase 3, multicenter, randomized, double blind, randomized controlled trial (NCT05797610, A Study to Evaluate the Efficacy and Safety of Sefaxersen (RO7434656) in Participants With Primary Immunoglobulin A (IgA) Nephropathy at High Risk of Progression), sponsored by Hoffmann-La Roche, designed to evaluate the efficacy and safety of Sefaxersen in adult IgAN patients [57].

## CONCLUSIONS

In this narrative review, we explored the outcomes achieved by RNAi therapies which represent a revolutionary approach in the treatment of rare and ultra-rare disorders and diseases in which kidney involvement may occur. These therapeutic strategies can selectively silence or downregulate a specific mRNA transcript. A set of innovative drugs has been approved by the FDA and/or the EMA, but some of them need to be validated via clinical trials in which a large number of patients with rare or ultra-rare diseases or disorders are enrolled. However, some of these promising new approaches are currently undergoing evaluation for use in more common diseases such as hypertension [58] and diabetes [59], which are more prevalent causes of kidney damage than rare and ultrarare diseases or disorders.

## Data Availability

No datasets were used.
